# Outcomes of 23 patients diagnosed with New Delhi metallo-beta-lactamase (NDM)-producing Klebsiella pneumoniae infection treated with ceftazidime/avibactam and aztreonam at a single center in Poland

**DOI:** 10.1007/s10096-024-04859-y

**Published:** 2024-05-29

**Authors:** Aneta Guzek, Zbigniew Rybicki, Dariusz Tomaszewski, Katarzyna Mackiewicz, Wiesław Piechota, Andrzej Chciałowski

**Affiliations:** 1 Section of Microbiology, Department of Laboratory Diagnostics, Military Institute of Medicine—National Research Institute, Warsaw, Poland; 2 Department of Anesthesiology and Intensive Therapy, Military Institute of Aviation Medicine, Warsaw, Poland; 3 Department of Laboratory Diagnostics, Military Institute of Medicine—National Research Institute, Warsaw, Poland; 4 Department of Infectious Diseases and Allergology, Military Institute of Medicine—National Research Institute, Warsaw, Poland; 5 Department of Anesthesiology and Intensive Therapy, Military Institute of Medicine—National Research Institute, Warsaw, Poland

**Keywords:** Avibactam, Ceftazidime drug combination, Aztreonam, Beta-lactamases, Klebsiella pneumoniae

## Abstract

**Purpose:**

Amongst all etiologic hospital-acquired infection factors, *K. pneumoniae* strains producing New Delhi metallo-β-lactamase (KP-NDM) belong to pathogens with the most effective antibiotic resistance mechanisms. Clinical guidelines recommend using ceftazidime/avibactam with aztreonam (CZA + AT) as the preferred option for NDM-producing Enterobacterales. However, the number of observations on such treatment regimen is limited. This retrospective study reports the clinical and microbiological outcomes of 23 patients with KP-NDM hospital-acquired infection treated with CZA + AT at a single center in Poland.

**Methods:**

The isolates were derived from the urine, lungs, blood, peritoneal cavity, wounds, and peritonsillar abscess. In microbiological analysis, mass spectrometry for pathogen identification, polymerase chain reaction, or an immunochromatographic assay for detection of carbapenemase, as well as VITEK-2 system, broth microdilution, and microdilution in agar method for antimicrobial susceptibility tests were used, depending of the pathogens’ nature. CZA was administered intravenously (IV) at 2.5 g every eight hours in patients with normal kidney function, and aztreonam was administered at 2 g every eight hours IV. Such dosage was modified when renal function was reduced.

**Results:**

KP-NDM was eradicated in all cases. Four patients (17.4%) died: three of them had a neoplastic disease, and one - a COVID-19 infection.

**Conclusion:**

The combination of CZA + AT is a safe and effective therapy for infections caused by KP-NDM, both at the clinical and microbiological levels. The synergistic action of all compounds resulted in a good agreement between the clinical efficacy of CZA + AT and the results of in vitro susceptibility testing.

## Introduction

Infections, particularly those categorized as hospital-acquired, pose a global challenge, propelled by demographic factors such as a growing population over 70, compromised immunity linked to conditions like cancer and diabetes, and travels to regions with lower epidemiological standards. However, the primary factor is the escalating resistance of bacteria to antimicrobials, particularly among Gram-negative bacilli [1, 2]. This challenge is aggravated by the insufficient development of new antibiotics capable of addressing the evolving antibiotic resistance mechanisms that bacteria, particularly *Klebsiella pneumoniae*, *Pseudomonas aeruginosa*, and *Acinetobacter baumannii*, are developing [3].

The breakthrough was the synthesis of carbapenems in the late 1970s, which to date are the primary weapon in treating infections caused by Enterobacterales possessing an extended-spectrum β-lactamases (ESBL) type resistance mechanism.

*Klebsiella pneumoniae*, one of the Enterobacterales family members, is commonly found in the oral cavity, nasal passages, skin, and gastrointestinal tract. The carriage rate of *K. pneumoniae* in the oral cavity varies from 5% in Western populations to 30% in Asia, and in the gastrointestinal tract, it ranges from 5 to 35% to even 60-70%, respectively. Although colonization is asymptomatic, carriage in the gastrointestinal tract may be a requisite for infection. *K. pneumoniae* is also a reservoir of antibiotic-resistance genes that can be transferred to other pathogens [4]. A significant proportion of such strains produce extended-spectrum β-lactamases. Those producing NDM belong to bacteria with the most effective antibiotic resistance mechanisms. The threat associated with the presence of KP-NDM is linked to its three characteristics: pathogen resistance to all or almost all available antibiotics, including colistin; ability to transfer resistance genes to other microorganisms (e.g., *E. coli*) through the localization of genes on plasmids and transposons; and high potential for spread within hospitals.

These characteristics limit the options for treating infections caused by KP-NDM. Colistin, gentamicin, tigecycline, and fosfomycin, used in the treatment of these infections, can lead to serious adverse effects, such as nephrotoxicity [5] and hepatotoxicity [6]. Karakonstantis et al. presented a therapeutic algorithm depending on the type of carbapenemase produced by carbapenem-resistant *K. pneumoniae* strains [7].

An unquestionable success of recent years has been the synthesis of new β-lactamase inhibitors like tazobactam, avibactam, vaborbactam, and relebactam, which, when combined with antibiotics like ceftalozane, ceftazidime, carbapenems have high efficacy against Enterobacterales capable of producing class A carbapenemases (*K. pneumoniae* carbapenemase [KPC], imipenem-hydrolyzing β-lactamase [IMI], *Serratia marcescens* enzyme [SME], Guiana extended-spectrum β-lactamase [GES], and class D (carbapenemase/oxacillinase [OXA]-48). However, class B carbapenemases so-called metallo-β-lactamases (MBLs: New Delhi metallo-β-lactamase [NDM], imipenemase metallo-β-lactamase [IMP], Verona integron-encoded metallo-β-lactamase [VIM] break down all β-lactamase inhibitors and combined antibiotics except aztreonam.

The guidelines of the European Society of Clinical Microbiology and Infectious Diseases (ESCMID) [8] and the Infectious Diseases Society of America (IDSA) [9] recommend the use of ceftazidime/avibactam with aztreonam (CZA + AT) as the preferred treatment option for Enterobacterales producing NDM-type metallo-β-lactamases. However, the number of observations on such treatment regimen in infections caused by Gram-negative bacteria capable of producing MBL is limited. The most extensive paper on this topic included 82 patients with bacteremia caused by Enterobacterales NDM. It showed the therapeutic superiority of CZA + AT over other combination therapies [10].

Among the references provided by the authors above, only four comparable papers with notably fewer cases were identified. In the 2022 review article on utilizing new antibiotics encompassing CZA + AT therapy, only one report addressed the clinical follow-up of such treatment [11]. The conclusion of the extensive review by Karakonstakis et al. [7], summarizing therapeutic options for infections with multidrug-resistant strains of *K. pneumoniae*, *P. aeruginosa*, and *A. baumannii*, emphasizes the need for further research on synergistic antibiotic combinations and their administration regimens. According to the authors of this study, it aligns with the necessity of extending in vitro assessment of the treatment efficacy of the clinical one.

Therefore, this study aimed to report the clinical and microbiological outcomes of 23 patients diagnosed with KP-NDM infection treated with CZA + AT at a single center in Poland.

## Materials and methods

### Study protocol

This paper is a retrospective study based on clinical material from the Military Medical Institute—National Research Institute in Warsaw, Poland, collected between June 2022 and November 2023. The study included 23 consecutive patients manifesting clinical symptoms of KP-NDM hospital-acquired infection.

### Bacterial isolates identification and susceptibility testing

#### Bacterial isolates

The 23 KP-NDM isolates were derived from the following biological materials: urine (*n* = 8), lower respiratory tract (*n* = 7), blood (*n* = 3), peritoneal cavity (*n* = 2), wounds (*n* = 2), peritonsillar abscess (*n* = 1).

#### Bacterial identification

The strains were identified as *K. pneumoniae* by Matrix-Assisted Laser Desorption Ionization-Time Of Flight mass spectrometry (MALDI-TOF) (VITEK MS, bioMérieux, France).

Bacterial colonies were sampled from blood agar plates using a plastic loop with a capacity of 1 µl and transferred onto disposable target plates (VITEK MS, bioMérieux). Subsequently, one microliter of matrix solution (α-cyano-4-hydroxycinnamic acid, VITEK MS CHCA) was added. After air drying at room temperature, the target plates were analyzed using a mass spectrometer (VITEK MS). The main spectral profiles of the isolates were obtained under standard identification settings (positive linear mode, 2000–20,000 Da) using the VITEK MS IVD database version 3.2, comprising 15,556 microbial strains covering 1,316 species. Calibration and quality control for each set of 16 samples were conducted using *E. coli* ATCC 8739. The VITEK MS’s software calculated the confidence value and percentage probability to reflect the concordance of the observed spectrum with its database. Confidence values above 99% were interpreted as high-confidence results, between 60 and 99% as low-confidence results, and below 60% as unidentified. All strains were identified as K. pneumoniae with a confidence value of > 99%.

#### Detection of carbapenemases

The presence and nature of carbapenemase determinants were assessed by molecular testing of bacterial isolates using either a polymerase chain reaction (PCR) based platform GeneXpert System (Cepheid, USA) or a lateral flow immunochromatographic assay (RESIST-5 OOKNV, CORIS, BioConcept, Belgium).

#### Antimicrobial susceptibility testing

Antimicrobial susceptibility tests were performed using the VITEK-2 automated system (bioMérieux, France), according to the manufacturer’s instructions, except for the colistin, fosfomycin, and cefiderocol determination. The minimal inhibitory concentration (MIC) of colistin was determined by micro-dilution in broth using the MICRONAUT MIC-Strip colistin (MMS) assay (Merlin Diagnostika GmbH, Germany). The MIC of fosfomycin was determined by microdilution method in Mueller Hinton Agar supplemented with 25 mg/L of glucose-6-phosphate using the AD Fosfomycin panel (Liofilchem®, Roseto degli Abruzzi, Italy), while the MIC of cefiderocol was determined by broth microdilution method using the ComASP® Cefiderocol panel, Liofilchem, Italy (Liofilchem®, Roseto degli Abruzzi, Italy).

The results were read according to the European Committee on Antimicrobial Susceptibility Testing (EUCAST) criteria [12].

#### Combination testing

The MIC test strip fixed-ratio method evaluated the synergy between CZA and AT (7–9). Respective MIC strips/scales were used to read MICs by placing them in each gradient’s position, and the FIC (Fractional inhibitory concentration) index was calculated. *E. coli* ATCC 25,922 was used as a quality control strain in all these experiments. The FIC was calculated and interpreted as described below:

- FIC of agent A = MIC of agent A combination / MIC of agent A alone;
- FIC of agent B = MIC of agent B in combination / MIC of agent B alone;
- Cumulative FIC = FIC of agent A + FIC of agent B.

Synergy is interpreted when the FIC index is ≤ 0.5, addition corresponds to > 0.5 to ≤ 1, indifference corresponds to the FIC index > 1 to ≤ 4, and antagonism when the FIC index is > 4.0 [13, 14].

### Antibiotic therapy

CZA was administered at 2.5 g every eight hours by intravenous (IV) infusion over 2 h in patients with normal kidney function. In patients with a creatinine clearance of 16–30 ml/min, CZA was administered at a dose of 0.75 g/0.1875 g every 12 h IV, and at lower clearance values, the dose was administered every 24 h. Aztreonam was administered at two grams every eight hours IV, while the antibiotic dose was halved in patients with a creatinine clearance of 10–20 ml/min.

### Patients’ evaluation

The patients’ condition was continuously monitored with clinical examination, biochemical and microbiological tests. The clinical assessment involved monitoring the level of consciousness, body temperature, respiratory system function, circulatory system function, renal function, and - if the patient’s condition or reported symptoms required - other parameters. Blood samples were regularly collected for biochemical tests to monitor the dynamics of inflammatory markers and other parameters and objectively assess the patients’ condition. Microbiological diagnostics were also conducted.

### Statistical analysis

The data were archived using Microsoft Excel. Statistical analysis was performed using R software version 4.3.1 (Vienna, Austria). Descriptive statistics were employed. Due to the relatively small sample size, the results are presented as the median and interquartile range.

## Results

### The general characteristics of the study patients

The general characteristics of analyzed patients are presented in Tables 1 and 2. Among the 23 investigated patients, a subgroup of eight individuals can be identified in whom the infection manifested as septic shock. These mentioned patients required aggressive pharmacotherapy, the administration of catecholamines, and mechanical ventilation. In the analyzed group, four patients (17.4%) succumbed to the illness. Among those who died, an additional complicating factor, independent of the infection, was a neoplastic disease (3 patients) and, in one case, COVID-19.

**Table 1:** The general characteristics of analyzed patients

Hospital ward	Type of infection	Gender (M/F)	Age (years)	Hospitalization time (days)	SOFA score	CZA + AT therapy (days)	Pathogen’s eradication (y/*n*)	Outcome (discharge/death)
(1)	(2)	(3)	(4)	(5)	(6)	(7)	(8)	(9)
Cardiology	Blood infection	2/0	55 (27)	55 (27)	3 (1)	10 (0)	2/0	2/0
	UTI	3/0	82 (6)	44 (15)	2 (0)	14 (2)	3/0	3/0
ENT	Peritonsillar abscess	1/0	53	19	1	10	1/0	1/0
Gastrology	Blood infection	1/0	88	23	6	10	1/0	0/1
	UTI	1/0	48	54	2	14	1/0	1/0
Hematology	Pneumonia	1/0	80	12	2	10	1/0	1/0
ICU	Pneumonia	5/0	57 (11)	52 (33)	6 (0)	10 (2)	5/0	5/0
Nephrology	UTI	3/1	61 (20)	19 (13)	4 (3)	10 (0)	4/0	2/2
Orthopedics	Wound infection	1/0	51	113	1	10	1/0	1/0
Rheumatology	Pneumonia	0/1	77	86	3	12	1/0	0/1
Surgery	Peritonitis	1/1	43 (10)	63 (35)	5 (2)	12 (2)	2/0	2/0
	UTI	1/0	50	39	2	10	1/0	1/0

Data in 4th, 5th, 6th and 7th column were presented as median and interquartile range

M – male, F – female, SOFA – sequential organ failure assessment, CZA – ceftazidime with avibactam, AT – aztreonam

UTI – urinary tract infection, ENT – ear, nose, and throat, ICU – intensive care unit

**Table 2:** Detailed characteristics of analyzed patients

Patient	Age, Gender	Ward of hospitalization	The reason for hospital admission (ICD10)	Duration of Hospitalization (days)	Type of Infection	Patient’s clinical condition			Biochemical results							CZA + AT therapy Duration (days)	Pathogen’s eradication	Outcome
			Comorbidities (ICD10)			Respiratory function	Circulation	SOFA Score (points)		CRP	PCT	WBC	Crea	GFR	Bili			
1	77, F	Rheumatology	R18	86	Pneumonia	Oxygen supplementation via face mask	Sufficient	3	Before therapy	10.4	0.38	8.45	1.4	39	0.3	12	Yes	Death
			I10, F00						After therapy	9.0	0.12	6.70	1.1	51	0.3			
2	66, M	ICU	I06	79	Pneumonia	Ventilatory support, FiO_2_ 0.5	DOB, EPI, NOR In iv infusion	6	Before therapy	16.5	2.28	12.1	0.4	> 90	0.5	12	Yes	Home discharge
			I06, I25, I48, E66, G47						After therapy	3.4	0.14	5.75	0.3	> 90	0.3			
3	33, F	Surgery	K28, K65, K56	97	Peritonitis	Ventilatory support, FiO_2_ 0.5	DOB, EPI, NOR In iv infusion	6	Before therapy	17.8	48.2	51.8	0.5	> 90	0.4	11	Yes	Home discharge
			E66						After therapy	8.8	0.12	11.08	0.5	> 90	0.2			
4	74, M	Nephrology	M31, N08	10	UTI	Sufficient	Sufficient	2	Before therapy	0.8	0.73	6.33	3.5	20	0.4	10	Yes	Home discharge
			N18						After therapy	0.5	0.23	5.86	3.2	18	0.4			
5	24, F	Nephrology	N18	21	UTI	Sufficient	Sufficient	2	Before therapy	2.1	0.8	6.03	3.1	19	0.1	10	Yes	Home discharge
			I10						After therapy	1.2	0.25	4.65	2.0	33	0.4			
6	66, M	Nephrology	A41, j96	48	UTI	Oxygen supplementation via face mask	NOR in IV infusion	5	Before therapy	5.2	0.88	17.8	4.1	12	0.1	10	Yes	Death
			I25, E11						After therapy	3.6	0.5	13.77	2.8	18	0.2			
7	71, M	Cardiology	I50	32	UTI	Sufficient	Sufficient	2	Before therapy	1.5	0.3	4.74	2.3	30	0.5	10	Yes	Home discharge
			N18						After therapy	1.1	0.11	3.49	1.9	37	0.7			
8	82, M	Aardiology	I50	62	UTI	Sufficient	Sufficient	2	Before therapy	5.7	0.7	6.58	1.9	39	1.1	14	Yes	Home discharge
			N18, i10, E11						After therapy	1.2	0.5	10.1	1.8	36	0.7			
9	52, M	Surgery	K63	28	Peritonitis	Sufficient	Sufficient	3	Before therapy	18.3	1.18	22.26	0.6	> 90	0.5	15	Yes	Home discharge
			D51						After therapy	2.3	0.36	14.22	0.4	> 90	0.2			
10	57, M	ICU	J96	43	Pneumonia	Ventilatory support, FiO_2_ 0.5	NOR in IV infusion	6	Before therapy	14.10	1.54	21.77	1.3	> 90	0.2	10	yes	Home discharge
			N17, G12						After therapy	4.16	0.43	11.75	0.6	> 90	0.3			
11	73, M	ICU	J96	76	Pneumonia	Ventilatory support, FiO_2_ 0.5	NOR in IV infusion	6	Before therapy	10.2	1.18	18.09	0.9	> 90	0.6	14	Yes	Home discharge
			E10, I10, I50						After therapy	1.2	0.36	5.17	0.6	> 90	0.3			
12	53, M	ENT	K71	19	Peritonsillar abscess	Sufficient	Sufficient	1	Before therapy	11.4	0.44	8.93	0.4	> 90	0.1	10	Yes	Home discharge
			K70, D51						After therapy	5.9	0.2	5.94	0.5	> 90	0.1			
13	80, M	Hematology	C91	12	Pneumonia	Sufficient	Sufficient	2	Before therapy	12.2	0.14	7.07	0.7	> 90	0.3	10	Yes	Home discharge
			-						After therapy	1.2	0.1	5.78	0.5	> 90	0.3			
14	50, M	Surgery	C60	39	UTI	Sufficient	Sufficient	2	Before therapy	19	0.09	9.67	1.5	53	0.5	10	Yes	Home discharge
			E66						After therapy	1.5	0.04	6.54	1.3	> 90	0.3			
15	48, M	Gastrology	K85	54	Blood	Sufficient	NOR in IV infusion	2	Before therapy	19.5	9.05	16.63	0.5	> 90	0.9	14	yes	Home discharge
			I10						After therapy	10.8	1.82	31.52	0.6	> 90	0.5			
16	56, M	Nephrology	N18	17	UTI	Sufficient	NOR in IV infusion	6	Before therapy	19.5	9.05	16.63	0.5	> 90	0.4	10	Yes	Death
			C18						After therapy	1.82	31.52	0.6	> 90	0.5	0.5			
17	88, M	Cardiology	I33	28	Blood	Sufficient	Sufficient	2	Before therapy	4.7	0.7	2.32	0.8	> 90	0.4	10	Yes	Home discharge
			E78						After therapy	0.38	5.57	0.6	> 90	0.3	0.38			
18	47, M	ICU	J96	32	Pneumonia	Ventilatory support, FiO_2_ 0.5	NOR in IV infusion	6	Before therapy	23.7	3.99	20.10	2.3	31	0.4	10	yes	Home discharge
			I50, N17, J15						After therapy	4.32	0.81	4.97	1.7	43	0.4			
19	55, M	ICU	J96	40	Pneumonia	Ventilatory support, FiO_2_ 0.5	NOR in IV infusion	6	Before therapy	21	2.92	23.36	2.3	31	3.3	10	Yes	Home discharge
			E66, I10, I50, E11, N18						After therapy	1.83	0.25	10.12	1	> 90	1.1			
20	51, M	Orthopedics	T84	113	Wound	Sufficient	Sufficient	1	Before therapy	4.3	0.07	9.05	1	> 90	0.3	10	Yes	Home discharge
			M86						After therapy	1.3	0.05	5.58	0.8	> 90	0.3			
21	73, M	Cardiology	J96	81	Blood	Ventilatory support, FiO_2_ 0.5	Sufficient	4	Before therapy	16.1	1.54	11.11	0.8	> 90	3.2	10	Yes	Home discharge
			K74, I10, K83						After therapy	15.4	0.94	9.25	0.4	> 90	0.4			
22	88, M	Gastrology	K92	23	UTI	Sufficient	Sufficient	2	Before therapy	15.4	0.94	9.25	0.4	> 90	0.4	10	Yes	Death
			C80, D64						After therapy	9.1	0.75	22.85	1.1	> 90	0.5			
23	83, M	Cardiology	I50	44	UTI	Sufficient	Sufficient	2	Before therapy	4.7	0.7	2.32	0.8	> 90	0.4	14	Yes	Home discharge
			I07						After therapy	1.3	0.05	5.58	0.8	> 90	0.3			

M – male, F – female, SOFA – sequential organ failure assessment, CZA + AT – ceftazidime/avibactam and aztreonam, CRP = C-reactive protein (mg/dL), PCT – procalcitonin (ng/mL), WBC – white blood cells (x10^9^/L), crea – creatinine (mg/dL), GFR – glomerular filtration rate (mL/minute/1.73m^2^), bili – bilirubine (mg/dL), ICU – intensive care unit, FiO2 – fraction of inspired oxygen, DOB – dobutamine, EPI – epinephrine, NOR – norepinephrine, UTI – urinary tract infection

ICD10 codes: A41 – sepsis, C18 – malignant neoplasm of colon, C60 – malignant neoplasm of penis, C80 – malignant neoplasm without specification of site, C91 – lymphoid leukemia, D51 – vitamin B12 deficiency anemia, D64 – other anemias, E10 – type 1 diabetes mellitus, E11 – type 2 diabetes mellitus, E66 – overweight and obesity, E78 – disorders of lipoprotein metabolism and other lipidemias, F00 – dementia in Alzheimer disease, G12 – spinal muscular atrophy and related syndromes, G47 – sleep disorders, I06 – rheumatic aortic valve disease, I07 – rheumatic tricuspid valve disease, I10 – essential (primary) hypertension, I25 – chronic ischemic heart disease, I33 – acute and subacute endocarditis, I48 – atrial fibrillation and flutter, I50 – heart failure, J15 – bacterial pneumonia, J96 – respiratory failure, K28 – gastrojejunal ulcer, K56 – paralytic ileus and intestinal obstruction without hernia, K63 – other diseases of intestine, K65 – peritonitis, K70 – alcoholic liver disease, K71 – toxic liver disease, K74 – fibrosis and cirrhosis of liver, K83 – other diseases of biliary tract, K85 – acute pancreatitis, K92 – other diseases of the digestive system, M31 – other necrotizing vasculopathies, M86 – osteomyelitis, N08 – glomerular disorders in diseases classified elsewhere, N18 – chronic kidney disease, R18 – ascites, T84 – complications of internal orthopedic prosthetic devices, implants and grafts

#### Blood laboratory tests

The C-reactive Protein (CRP), procalcitonin (PCT), and white blood cell count (WBC) were monitored to follow the dynamics of the infection. In all cases, a decrease in their levels from baseline values was observed during treatment. Creatinine and eGFR levels were determined to guide antibiotic dosage, whereas bilirubin levels were measured to reduce the antibiotics’ hepatotoxicity.

#### Microbiological investigations

Bacterial eradication from the site of infection occurred in all cases.

Table 3 contains data on those antibiotics to which antibiotic susceptibility of KP-NDM has been achieved to a greater or lesser extent. The bacterial strains from all the patients were 100% susceptible to cefiderocol. The microbiological results showed a 46% susceptibility to fosfomycin and trimethoprim/sulfamethoxazole, 31% to colistin and gentamicin, and 8% to amikacin and gentamicin.

**Table 3:** In vitro antibiotic susceptibility for 23 *K. pneumoniae* NDM isolates

Antimicrobial agent	Range µg/mL	Susceptible (S) %	Resistant (*R*) %
Amikacin	2–32	8	92
Amoxicillin/clavulanic acid	> 16	-	100
Aztreonam	64 to ≥ 256	-	100
Cefiderocol	0.008-128	100	-
Cefepime	> 16	-	100
Cefotaxime	> 32	-	100
Ceftazidime	> 32	-	100
Ceftazidime/Avibactam	≥ 256	-	100
Cefuroxime	> 32	-	100
Ciprofloxacin	> 2	-	100
Colistin	0.0625 to 64	31	69
Fosfomycin	0.25 to 256	46	54
Gentamicin	≤ 1 to > 8	31	69
Imipenem	> 8 to > 32	-	100
Meropenem	> 8 to > 32	-	100
Piperacillin/tazobactam	> 64	-	100
Tigecycline	4	-	100
Tobramycin	< 1 to > 8	8	92
Trimethoprim/sulfamethoxazole	≤ 1 to > 8	46	54

MIC values of CZA + AT and results of the Etest MIC: MIC ratio method is displayed in Table 4. All the tested isolates were resistant to ceftazidime/avibactam and aztreonam separately. The combination (MIC test strip fixed-ratio method) of ceftazidime/avibactam and aztreonam displayed synergy against all the tested isolates with FIC of < 0.5, and none of the isolates had additive or antagonistic responses by this method (Table 4).

**Table 4:** MIC values and synergistic activity assessed via the Etest fixed-ratio method

Patients	MIC CZA	MIC AT	MIC CZA + AT	Σ FIC	Synergy Interpretation
1	256	256	0.25	0.002	S
2	256	64	0.19	0.004	S
3	256	256	0.5	0.004	S
4	256	96	0.38	0.005	S
5	256	256	0.5	0.004	S
6	256	128	0.19	0.002	S
7	256	256	0.38	0.002	S
8	256	64	0.25	0.005	S
9	256	256	0.5	0.004	S
10	256	96	0.38	0,005	S
11	256	256	0.5	0.004	S
12	256	128	0.25	0.003	S
13	256	96	0.38	0.005	S
14	256	256	0.25	0.002	S
15	256	256	0.38	0.002	S
16	256	128	0.19	0.002	S
17	256	256	0.25	0.002	S
18	256	96	0.38	0.005	S
19	256	256	0.38	0.002	S
20	256	128	0.19	0.002	S
21	256	96	0.38	0.005	S
22	256	256	0.38	0.002	S
23	256	256	0.25	0.002	S

AT- aztreonam; CZA - ceftazidime-avibactam; Σ FIC - cumulative fractional inhibitory concentration; MIC – minimal inhibitory concentration; S - synergistic

## Discussion

In our study, all patients with symptoms of infection caused by KP-NDM treated with CZA + AT and pathogen eradication, defined as a negative result in microbiological testing, were achieved. This confirms the clinical effectiveness of such antibiotic therapy, even in patients with symptoms of septic shock. Combined treatment with CZA + AT for infections caused by Enterobacterales is recommended by ESCMID [8] and IDSA [9].

The benefits of such a combination stem from the fact that MBLs do not hydrolyze aztreonam, while CZA remains active against bacteria that can produce ESBLs and non-NDM carbapenemases.

Ceftazidime is a wide-spectrum cephalosporin, while avibactam is a β-lactamase inhibitor. Avibactam exhibits activity against all types of ESBLs, KPC, OXA48, and AmpC, but not MBLs (NDM, VIM, IMP). The ceftazidime/avibactam combination is effective against ESBLs, KPC, OXA-48, and AmpC, thereby restoring the efficacy of ceftazidime against Gram-negative bacteria. However, it does not work against MBL strains. Aztreonam is inactivated by ESBLs, KPC, and other cephalosporinases but remains stable against hydrolysis by MBLs. Thus, adding aztreonam to CZA enhances its activity by protecting aztreonam from the attack of ESBLs [15, 16].

In addition, the beneficial effect of such a combined set of antibiotics is the low MIC values of CZA + AZT shown in Table 3. Comparable findings were noted in a study encompassing 21 cases of *K. pneumoniae* NDM; specifically, in 8 samples, the MIC was below 0.13 µg/mL, while in the remaining cases, it measured 0.25 µg/mL [17]. However, this was only an in vitro study without assessing clinical outcomes. The same remark applies to the paper by Jayol et al. [15], who reported in vitro efficacy of the combination of ceftazidime-avibactam and aztreonam against isolates of carbapenemase-producing Klebsiella pneumoniae. This combination also proved effective in treating NDM-1-producing *Klebsiella pneumoniae* bacteremia in a neutropenic patient [18].

A comparison of the clinical efficacy of the combination of ceftazidime-avibactam and aztreonam versus treatment with colistin with fosfomycin and tigecycline or tigecycline with aminoglycosides or fosfomycin shows that the 30-day mortality rate for CZA + AT treatment was 19.2% and for other antibiotic combinations was much higher 44%. The most increased 30-day mortality was observed in patients treated with colistin-based regimens (59.3%) [10].

From our observations, the mortality rate was 17.4%. Four out of the analyzed patients died; three of them had a neoplastic disease, and one had a COVID-19 infection. This represents a lower mortality rate than in the Sree et al. [16] (29.9%) analysis but higher than in the study by Zhang et al. [19] (7.1%), although the latter analyzed hematologic patients.

The decrease in laboratory indicators of infection in our patients and the eradication of bacteria from the sites of infection show the effectiveness of the combination therapy.

Although the optimal pharmacokinetics/pharmacodynamics (PK/PD) values for the CZA + AT combination are not precisely defined, it can be assumed that these drugs have good tissue penetration in inflammatory conditions, especially when CZA + AT was given as a two-hour intravenous infusion, which ensures good concentration at the site of infection. Despite nearly half of the cases affecting the urinary tract, where antibiotics attain high concentrations, remarkable clinical efficacy was also observed in lung and abdominal infections, where the illness progressed to severe sepsis. This observation indirectly underscores the therapeutic effectiveness of the CZA + AT combination regimen.

No adverse clinically manifested side effects associated with the antibiotics used were observed.

Our study has some limitations. First of all, the number of observed patients is not large. It may limit our conclusions. Also, due to ethical considerations in line with current recommendations, the study lacks a comparative element with the most widely used antibiotic, colistin. In our analysis, the determination of MIC values for fosfomycin and cefiderocol was performed with a non-reference method. According to the EUCAST website communication [20], there is still no recommendation regarding an entirely reliable method for determining the susceptibility of microorganisms to cefiderocol. In our study, we employed, commercial tests that, according to the manufacturer, were validated by reference methods and held IVD certification.

Nevertheless, the unequivocal favorable results of the treatment entitle us to conclude that combined CZA + AT treatment is a safe, effective option for treating infections caused by *K. pneumoniae* having the ability to produce metallo-β-lactamases.

## Conclusion

Our results support the conclusion that combined treatment of ceftazidime/avibactam and aztreonam is a safe and effective therapeutic option for infections caused by *K. pneumoniae* capable of producing metallo-β- lactamases, both at the clinical and microbiological levels. The synergistic action of all compounds resulted in an excellent agreement between the clinical efficacy of ceftazidime/avibactam and aztreonam and the results of in vitro susceptibility testing of K. pneumoniae.

## Data Availability

No datasets were generated or analysed during the current study.
